# Phylogenetic Analyses of *RdRp* Region and *VP1* Gene in Human Norovirus Genotype GII.17[P17] Variants

**DOI:** 10.3390/microorganisms14040770

**Published:** 2026-03-28

**Authors:** Fuminori Mizukoshi, Yen Hai Doan, Asumi Hirata-Saito, Hiroyuki Tsukagoshi, Takumi Motoya, Ryusuke Kimura, Tomoko Takahashi, Yuriko Hayashi, Yuki Matsushima, Kei Miyakawa, Naomi Sakon, Kenji Sadamasu, Kazuhisa Yoshimura, Nobuhiro Saruki, Yoshiyuki Suzuki, Masashi Uema, Kosuke Murakami, Kazuhiko Katayama, Akihide Ryo, Tsutomu Kageyama, Hirokazu Kimura

**Affiliations:** 1 Department of Bioinformatics and Integrative Omics, National Institute of Infectious Diseases, Japan Institute for Health Security, Musashimurayama-shi 208-0011, Tokyo, Japan; mizukoshi.f@jihs.go.jp (F.M.);; 2 Department of Diagnostic Testing and Technology Research, National Institute of Infectious Diseases, Japan Institute for Health Security, Musashimurayama-shi 208-0011, Tokyo, Japan; doan.y@jihs.go.jp; 3 Department of Microbiology, Tochigi Prefectural Institute of Public Health and Environmental Science, Utsunomiya-shi 329-1196, Tochigi, Japan; 4 Department of Health Science, Gunma Prefectural Institute of Public Health and Environmental Sciences, Maebashi-shi 371-0052, Gunma, Japan; 5 Department of Virology, Ibaraki Prefectural Institute of Public Health, Mito 310-0852, Ibaraki, Japan; 6 Iwate Prefectural Research Institute for Environmental Sciences and Public Health, Morioka-shi 020-0857, Iwate, Japan; 7 Department of Health Science, Graduate School of Health Sciences, Gunma Paz University, Takasaki-shi 370-0006, Gunma, Japan; hayashi@paz.ac.jp; 8 Faculty of Medical Science and Technology, Gunma Paz University, Takasaki-shi 370-0006, Gunma, Japan; 9 Department of Diagnostic Testing and Technology Research, National Institute of Infectious Diseases, Japan Institute for Health Security, Shinjuku-ku, Tokyo 162-8640, Japan; 10 Influenza Research Center, National Institute of Infectious Diseases, Japan Institute for Health Security, Musashimurayama-shi 208-0011, Tokyo, Japan; 11 Department of Microbiology, Graduate School of Medicine, Yokohama City University, Yokohama 236-0004, Kanagawa, Japan; 12 Department of Microbiology, Osaka Institute of Public Health, Osaka 537-0025, Japan; 13 Department of Microbiology, Tokyo Metropolitan Institute of Public Health, 3-24-1 Hyakunincho, Shinjuku-ku, Tokyo 169-0073, Japan; 14 Tokyo Metropolitan Institute of Public Health, 3-24-1 Hyakunincho, Shinjuku-ku, Tokyo 169-0073, Japan; 15 Gunma Prefectural Institute of Public Health and Environmental Sciences, Maebashi-shi 371-0052, Gunma, Japan; 16 Division of Biological Science, Department of Information and Basic Science, Graduate School of Science, Nagoya City University, Nagoya-shi 467-8501, Aichi, Japan; yossuzuk@nsc.nagoya-cu.ac.jp; 17 Division of Biomedical Food Research, National Institute of Health Sciences, 3-25-26, Tonomachi, Kawasaki-ku, Kawasaki 210-9501, Kanagawa, Japan; 18 Laboratory of Viral Infection Control, Ōmura Satoshi Memorial Institute, Graduate School of Infection Control Sciences, Kitasato University, 5-9-1, Shirogane, Minato-ku, Tokyo 108-8641, Japan; katayama@lisci.kitasato-u.ac.jp; 19 Advanced Medical Science Research Center, Gunma Paz University, Takasaki-shi 370-0006, Gunma, Japan

**Keywords:** norovirus, genotype GII.17[P17], *RdRp* region and *VP1* gene, phylogenomic

## Abstract

In this study, we investigated the long-term evolutionary dynamics of human norovirus GII.17[P17] using the *RNA-dependent RNA polymerase* (*RdRp*) region and the *VP1* capsid gene, integrating phylogenetics, time-scaled inference, phylodynamics, and structure-based analyses. Maximum-likelihood phylogenies of both genomic regions consistently resolved four major clades (Clades 1–4). *VP1* patristic-distance distributions indicated higher within-clade diversity in the phylogenetically basal Clades 1 and 3, whereas Clades 2 and 4 showed lower diversity, consistent with recent demographic expansion. Similarity-plot analysis identified pronounced variability in the VP1 P2 domain, while the S and P1 domains remained comparatively conserved, supporting P2 as the primary hotspot of diversification. Bayesian time-scaled analyses estimated the most recent common ancestor around 1993 (*VP1*) and 2000 (*RdRp*) and revealed two major lineages (Clade 1/2 and Clade 3/4), with the split between Clades 3 and 4 occurring around 2016–2017. Bayesian skyline plots showed a marked increase in effective population size after 2013, and substitution-rate estimates indicated faster evolution in *VP1* than in *RdRp*, with higher *VP1* rates in the Clade 3/4 lineage than in Clade 1/2. Capsid dimer modeling further mapped high-confidence conformational B-cell epitopes and positively selected residues predominantly to the distal surface of P2, with broadly conserved spatial patterns across clades. Compared with the Clade 1 reference (Kawasaki323), Clade 2 accumulated numerous P2 substitutions, whereas Clades 3 and 4 retained fewer changes and remained closer to Clade 1 at the amino-acid level. Together, these results suggest lineage turnover within GII.17[P17] driven by constrained diversification at the P2 surface, potentially contributing to the recent predominance of the Clade 3/4 lineage.

## 1. Introduction

Human norovirus (HuNoV) is a non-enveloped virus with a non-segmented, positive-sense single-stranded RNA genome and is classified within the family *Caliciviridae*, genus *Norovirus*, species *Norovirus norwalkense* [1,2,3]. Based on the nucleotide sequence of the major capsid protein gene (*VP1*), noroviruses are classified into multiple genogroups (GI–GIX), of which GI and GII predominantly infect humans [3,4]. Each genogroup is further subdivided into genotypes, with GII.4 having caused repeated global epidemics through the emergence of antigenically distinct variants that drive successive epidemic waves [5,6,7,8]. Frequent recombination events occur at the junction of ORF1 and ORF2, generating recombinant strains that are designated using a dual nomenclature combining the *RNA-dependent RNA polymerase* (*RdRp*) genotype and the *VP1* genotype (e.g., GII.4[P16]).

Over the past decade, HuNoV GII infections have been dominated by several major genotypes, including GII.2, GII.4, and GII.17 [9,10,11,12]. Clinically, GII.2, GII.4, and GII.17 infections generally manifest as acute gastroenteritis with vomiting, diarrhea, nausea, and abdominal pain. Although some genotype-related differences in clinical presentation have been reported, current evidence indicates that these major genotypes share broadly similar clinical features [13,14]. Among these, GII.4 has maintained long-term global predominance through continuous antigenic drift and variant replacement [9,15]. In parallel, GII.17 has demonstrated substantial epidemic potential, most notably during its rapid global expansion in the mid-2010s, followed by intermittent periods of re-emergence [10,16]. Although GII.4 exhibits more sustained and widespread circulation, GII.17 has periodically increased in prevalence and, in some regions, temporarily rivaled GII.4 [10]. These two genotypes have played central roles in shaping the global epidemiological landscape of norovirus GII over the past ten years [12].

Following the emergence of SARS-CoV-2, the circulation of GII.17 declined markedly [17], likely reflecting the widespread implementation of non-pharmaceutical interventions that reduced enteric virus transmission. However, recent surveillance data indicate that GII.17 has re-emerged and caused renewed outbreaks since 2024 [18]. This renewed activity raises important questions regarding the drivers of GII.17 resurgence, as changes in host behavior alone may not fully explain its re-establishment in the post-COVID-19 period. Despite its epidemiological significance, the mechanisms underlying the decline, persistence, and re-emergence of GII.17 remain poorly understood.

Given the sustained predominance of GII.4 and GII.17 and their contrasting epidemic patterns, elucidating the molecular evolutionary dynamics of norovirus GII is critical. While changes in population immunity and viral genetic diversity have been proposed as contributing factors, previous studies have largely focused on outbreak descriptions or short-term phylogenetic analyses, leaving long-term evolutionary processes such as lineage turnover, selective pressures, and genetic continuity across epidemic phases insufficiently explored. In this study, we conducted a comprehensive molecular evolutionary analysis focusing on the GII.17[P17] genotype, which represents a genetically coherent lineage suitable for examining evolutionary trajectories spanning epidemic expansion, decline, and resurgence. By integrating time-resolved phylogenetic reconstruction with sequence-based analyses, we aimed to characterize evolutionary rates, lineage dynamics, and amino acid changes potentially associated with epidemic fitness. Our findings provide new insights into the molecular basis of norovirus persistence and re-emergence, contributing to a deeper understanding of norovirus evolutionary ecology and informing future surveillance and control strategies.

## 2. Materials and Methods

### 2.1. Viral Genome Sequencing by Next-Generation Sequencing

To investigate the molecular evolution of the *VP1* and *RNA-dependent RNA polymerase* (*RdRp*) genes of norovirus GII.17[P17] strains, whole-genome sequencing was performed on 18 randomly selected strains of this genotype detected in Japan during the 2023–2025 seasons using next-generation sequencing (NGS), as described previously [19]. Briefly, whole or near-complete viral genomes were generated using an RNA-based sequencing approach. DNA libraries were prepared from extracted total RNA derived from human clinical samples collected from patients with acute gastro-enteritis caused by NoV infection using the NEBNext Ultra II RNA Library Prep Kit for Illumina together with the NEBNext Multiplex Oligos for Illumina (96 Unique Dual Index Primer Pairs) (New England Biolabs, Ipswich, MA, USA), following the manufacturer’s instructions. Extracted RNA was fragmented and primed with random primers, followed by first- and second-strand complementary DNA (cDNA) synthesis. The resulting cDNA fragments were subjected to end repair and dA-tailing, ligated with adapters containing unique index primers, amplified by 15 cycles of PCR, and purified using AMPure XP beads (Beckman Coulter, Indianapolis, IN, USA). Library quality and fragment size distribution were assessed using a 4150 TapeStation system with D1000 ScreenTape (Agilent Technologies, Santa Clara, CA, USA), and library concentrations were determined using a Qubit 2.0 Fluorometer in combination with the Qubit dsDNA HS Assay Kit (Thermo Fisher Scientific, Waltham, MA, USA). Paired-end sequencing (2 × 151 bp) of pooled libraries was performed on an Illumina NextSeq platform using the NextSeq 1000/2000 P1 XLEAP-SBS Reagent Kit (300 cycles) (Illumina, San Diego, CA, USA).

Sequence data were analyzed using CLC Genomics Workbench v25 (QIAGEN, Hilden, Germany). Raw reads were initially assembled into contigs using the De Novo Assembly tool, and norovirus-related contigs were identified using the Annotate with BLAST 1.2 tool against reference norovirus sequences. In parallel, reads were mapped to GII.17[P17] reference genomes using the Map Reads to Reference tool. Final GII.17[P17] genome sequences were generated by integrating consensus sequences derived from both de novo assembly and reference-based mapping approaches.

The nucleotide sequences of the 18 GII.17[P17] strains determined in this study have been deposited in the GenBank database under accession numbers LC918507–LC918524 (Supplementary Table S1).

The strains used in this study were detected through public health-based laboratory diagnostic testing conducted under the National Epidemiological Surveillance of Infectious Diseases (NESID) Program, in accordance with the Act on the Prevention of Infectious Diseases and Medical Care for Patients with Infectious Diseases (Infectious Diseases Control Law) or Food Sanitation Act of Japan. Under this statutory surveillance framework, the collection of clinical specimens from patients for public health purposes does not require written informed consent, nor does it require an opt-out procedure. However, when specimens are used for purposes other than those stipulated under the NESID framework, such use should be conducted in accordance with separately established regulations, including the Ethical Guidelines for Medical and Health Research Involving Human Subjects. In compliance with this legal provision, a comprehensive ethical review of the present study was conducted by the National Institute of Infectious Diseases Ethics Committee, and formal approval was obtained (Approved No. 1887 on 9 December 2025). All research procedures were performed in accordance with the approved protocol and the ethical standards set forth by the Committee. In addition, verbal informed consent was obtained from the participants or their legal guardians. Because the specimens were anonymized and used retrospectively, the requirement for written informed consent was waived by the institutional ethics committee.

### 2.2. Retrieval of Publicly Available Norovirus Sequences from Databases

To analyze the molecular evolution of NoV GII.17[P17], publicly available genome sequences were retrieved from the NCBI Virus database (https://www.ncbi.nlm.nih.gov/labs/virus/vssi/#/; last accessed on 24 December 2025). Database searches were conducted using the queries “Norovirus GII, taxid: 122929 (restricted to Genotype GII.17)” and “Norovirus GII.17, taxid: 552592”, and all available whole-genome sequences were downloaded. These sequences were subsequently genotyped using the Norovirus Typing Tool (version 2.0) (https://mpf.rivm.nl/mpf/typingtool/norovirus/) (accessed on 24 December 2025) to confirm classification as NoV GII.17[P17] [4]. Sequences containing ambiguous nucleotides (e.g., N, Y, M, or R), as well as those lacking information on collection year or geographic origin, were excluded from the dataset. In addition, among samples detected within the same year, sequences showing 100% nucleotide identity were removed to avoid redundancy. Details of the final dataset are summarized in Supplementary Table S2.

Together with the genome sequences generated by NGS in this study, the total number of samples included in the molecular evolutionary analyses is shown in Table 1. Furthermore, Supplementary Figure S1 illustrates the annual distribution of detected strains and the temporal changes in the number of sequences analyzed for each year.

The sequence data of AB684681 (Hu/GII/27-3/Tokyo/1976/JPN), which represents the NoV GII.17[P17] prototype strain, were used as the reference sequence.

### 2.3. Maximum Likelihood Phylogenetic Analysis

Multiple sequence alignments of the nucleotide sequences or amino acid sequences were generated using MAFFT version 7.520 [20]. Maximum likelihood (ML) phylogenetic analyses were performed with IQ-TREE version 3.0.1, applying the ultrafast bootstrap approximation with 1000 replicates and the SH-like approximate likelihood ratio test (SH-aLRT) to assess branch support [21]. The best-fitting nucleotide substitution model was automatically selected using ModelFinder [22], as implemented in IQ-TREE. The resulting phylogenetic trees were visualized using FigTree version 1.4.

Clades were defined based on the overall tree topology, branch lengths, and bootstrap support values inferred from the ML phylogenetic analysis.

### 2.4. Assessment of Temporal Signal

Temporal signal was assessed before Bayesian time-scaled phylogenetic analysis by root-to-tip regression using TempEst version 1.5.3 [23]. Maximum-likelihood trees inferred from the *RdRp* region and *VP1* gene datasets were used as input, and sampling years were assigned to each sequence. In addition, separate assessments were performed for the *VP1* gene datasets grouped as Clade 1 and Clade 2, Clade 3 and Clade 4, respectively. The relationship between sampling time and root-to-tip divergence was evaluated using the R^2^ value.

### 2.5. Phylogenetic Distance Analysis

Phylogenetic distances within clades of NoV GII.17[P17] were quantified as patristic distances using the Newick-formatted trees derived from the maximum likelihood (ML) analysis described above. Patristic distances were calculated using the Patristic program [24]. Violin plots were generated using Orange Data Mining version 3.35 [25] to visualize the distribution of distances.

Statistical analyses were performed using GraphPad Prism version 7 (GraphPad Software, La Jolla, CA, USA). Comparisons between groups were conducted using an unpaired *t*-test with Welch’s correction. Statistical significance was defined as *p* < 0.0001.

### 2.6. Similarity Plot Analysis

To evaluate the conservation and nucleotide-level similarity of aligned *VP1* gene sequences of NoV GII.17[P17], pairwise similarity analyses were performed using SimPlot++ V1.3 [26]. A prototype strain detected in 1976 (AB684681: Hu/GII/27-3/Tokyo/1976/JPN) was used as the reference sequence. Nucleotide similarity was calculated based on the Kimura two-parameter model, using a sliding window size of 200 nucleotides and a step size of 20 nucleotides.

### 2.7. Time-Scaled Phylogenetic Analysis and RdRp-Based Discrete Trait Inference

To evaluate the molecular evolution of NoV GII.17[P17] strains, time-scaled phylogenetic trees were inferred for the *RdRp* region and the *VP1* gene using a Bayesian Markov chain Monte Carlo (MCMC) implemented in the BEAST package (version 2.7.7) [27]. In addition, strains for which *RdRp* and *VP1* sequences were available as paired data were selected. For these strains, clade assignments defined on the basis of the *RdRp* phylogeny were incorporated as discrete traits, and time-scaled phylogenetic analyses based on the *VP1* gene were subsequently performed.

First, the most appropriate nucleotide substitution model for each dataset was estimated and selected using the jModelTest2 program [28]. Next, combinations of six molecular clock models (strict clock, random local clock, optimized relaxed clock, relaxed clock exponential, relaxed clock log normal, and fast relaxed clock log normal) and two tree priors (coalescent constant population, coalescent exponential population, and coalescent Bayesian Skyline) were evaluated using path sampling and stepping-stone sampling (PS/SS) to identify the best-fitting model configuration [29].

Based on the optimal model combinations summarized in Supplementary Table S3, MCMC phylogenetic analyses were conducted using BEAST2 (version 2.7.7). MCMC chains were run for 300 or 500 million steps, with parameters sampled every 1000 steps. Convergence was assessed using Tracer version 1.7.2 [30], and effective sample size (ESS) values greater than 200 were considered indicative of adequate convergence. The final ESS values are summarized in Supplementary Table S4.

After discarding the initial 10% of samples from each MCMC run as burn-in, maximum clade credibility (MCC) trees were generated using TreeAnnotator version 2.7.6, included in the BEAST package. The resulting Bayesian phylogenetic trees were visualized using FigTree version 1.4, and 95% highest posterior density (HPD) intervals were calculated for all internal nodes.

### 2.8. Bayesian Skyline Plot Analysis

To evaluate the phylodynamic characteristics of NoV GII.17[P17] strains and each defined clade, Bayesian Skyline Plot (BSP) analyses were performed using BEAST software (version 2.7. 7) [27], and temporal changes in the effective population size were inferred based on the *RdRp* region and the *VP1* gene.

For each dataset, the optimal nucleotide substitution model was selected using the jModelTest2 program, as described above. The Bayesian Skyline model was specified as the coalescent tree prior, and the most appropriate molecular clock model was selected from six candidate models using path sampling and stepping-stone sampling (PS/SS), as described previously. The optimal combinations of substitution, clock, and coalescent models are summarized in Supplementary Table S3. MCMC chains were run for 300 or 600 million steps, with parameters sampled every 1000 steps. Visualization of the BSP and estimation of 95% highest posterior density (HPD) intervals were performed using Tracer version 1.7.2 [30].

In addition, molecular evolutionary rates were estimated for each dataset based on the optimal models selected as described above. Statistical comparisons of the estimated evolutionary rates among groups were performed using GraphPad Prism version 7 (GraphPad Software, La Jolla, CA, USA) with the Kruskal–Wallis test. Statistical significance was defined as *p* < 0.0001.

### 2.9. Three-Dimensional Structure Prediction of the Capsid (VP1) Protein

To compare the three-dimensional (3D) structures of the Capsid protein among subtypes, 3D structural models of Capsid were predicted using LocalColabFold (version 1.5.3) installed on a local computer [31]. For NoV GII.17[P17], Capsid protein models were constructed using representative strains selected from each defined clade. The amino acid sequences used for 3D structure prediction were trimmed by removing 11 amino acids from the N-terminus and 9 amino acids from the C-terminus, based on structural information available in the Protein Data Bank (PDB).

First, multiple sequence alignment files (a3m files) were generated in a local environment using uniref30 (2302) as the uniref database, PDB100 (230517) as the template database, and colabfold_envdb (202108) as the environmental sequence database. Second, structure prediction was performed with the following flags enabled: “--amber”, “--templates”, and “--use-gpu-relax”. The number of prediction recycles was set to 30. For each sequence, five prediction models were generated by LocalColabFold. The optimal model was selected by jointly considering the predicted local distance difference test (pLDDT), template modeling (TM) score, and root mean square deviation (RMSD). Finally, the selected models were visualized using UCSF ChimeraX (version 1.7.1) [32].

### 2.10. Structure-Based Conformational B-Cell Epitope Prediction

Conformational B-cell epitopes were predicted using the constructed three-dimensional (3D) structural models of the Capsid protein. Epitope prediction was evaluated using five independent methods: DiscoTope 3.0 (high-confidence cutoff score of 1.50; recall up to ~30%) [33], ElliPro (cutoff values of 0.5) [34], EpiGraph [35], SEPPA 3.0 (cutoff values of 0.089) [36], and SEMA (cutoff values of 0.76) [37]. Amino acid residues predicted as epitopes by four or more of these five methods were defined as conformational B-cell epitopes.

For each amino acid residue, the number of prediction methods identifying the residue as an epitope was visualized as a heatmap. Heatmaps were generated using Orange Data Mining (version 3.35) [25].

In addition, the predicted B-cell epitope residues were mapped onto each Capsid structural model and color-coded using UCSF ChimeraX (version 1.7.1) [32].

### 2.11. Selective Pressure Analyses

Site-specific selective pressures acting on the *RdRp* region and the *VP1* gene were assessed by estimating nonsynonymous (dN) and synonymous (dS) substitution rates at each codon using the Datamonkey web server (https://www.datamonkey.org/) (accessed on 12 January 2026) [38]. To identify sites under purifying (negative) selection, three complementary methods were applied: single-likelihood ancestor counting (SLAC) [39], fixed effects likelihood (FEL) [39], and fast unconstrained Bayesian approximation (FUBAR) [40].

In contrast, episodic positive selection was evaluated using the mixed effects model of evolution (MEME) [41], which is capable of detecting site-specific diversifying selection acting on a subset of lineages. For SLAC, FEL, and MEME analyses, statistical significance was defined as *p* < 0.05, whereas support for selection in FUBAR was inferred from a posterior probability greater than 0.9.

### 2.12. Recombination Analysis

Recombination analysis was performed using aligned nucleotide sequences spanning from the start codon of the *RdRp* region to the stop codon of *VP1* gene. The final dataset used for recombination screening consisted of 192 GII.P17-GII.17 sequences. To screen for recombination signals within the analyzed GII.P17-GII.17 strains, the dataset was analyzed using RDP5 with multiple detection methods, including RDP, BootScan, GENECONV, MaxChi, Chimaera, SiScan, and 3SEQ [42]. In addition, similarity plot and bootscan analyses were performed using SimPlot++ to visually assess possible mosaic structures [26]. Six query sequences were selected, including four representative strains from Clades 1–4 (Clade 1; AB983218/JPN/2014, Clade 2; LC037415/JPN/2015, Clade3; LC918510(IBR2024012)/JPN/2024, and Clade 4; LC918512(IBR2025014)/JPN/2025) and two intermediate strains (Clade 1/3; PQ373125/NLD/2016 and Clade 3/4; PV784273_DEU_2022). For these analyses, clade consensus sequences from Clades 1–4 were used as references, and candidate query strains were compared across the alignment spanning the ORF1/ORF2 junction.

### 2.13. Median-Joining Network Analysis of the VP1 Gene

To further assess the geographic distribution of human norovirus GII.17[P17] lineages, a median-joining network analysis was performed based on the *VP1* gene. *VP1* sequences with available country or region of origin were extracted from the dataset used for phylogenetic analysis. The network was constructed using PopART version 1.7 with the median-joining algorithm [43]. Sequences were color-coded according to country or region of origin.

## 3. Results

### 3.1. Maximum Likelihood Phylogenetic Analysis of the RdRp Region and VP1 Gene of NoV GII.17[P17]

Maximum likelihood (ML) phylogenetic trees of the NoV GII.17[P17] genotype were constructed based on the full-length nucleotide sequences of the *RdRp* region and the *VP1* gene (Supplementary Figure S2). The phylogenetic trees inferred from the *RdRp* and *VP1* sequences exhibited overall similar topologies.

Based on tree topology, branch lengths, bootstrap support values, and the year of detection for each strain, the GII.17[P17] sequences were classified into four major clades, designated Clade 1 through Clade 4. In addition, two strains located at phylogenetically more ancestral positions relative to Clade 3 were identified and were classified as intermediate Clade 1/3, as they occupied positions between Clade 1 and Clade 3. Likewise, strains located between Clade 3 and Clade 4 were defined as intermediate Clade 3/4.

The temporal distribution of each clade is shown in Supplementary Figure S1. Following the detection of Clade 1, which included the Kawasaki323 strain, Clade 2—represented by the Kawasaki308 strain—was detected as the predominant lineage over a certain period. Clade 3, which emerged after the COVID-19 pandemic, occupied a phylogenetically distinct position from those of Clade 1 and Clade 2. Furthermore, since 2023, Clade 4, which was phylogenetically more distant from Clade 3, has been detected and is currently recognized as the predominant circulating lineage. These patterns were consistently observed in analyses based on both the *VP1* gene and the *RdRp* region.

### 3.2. Temporal Signal in the Dataset

Root-to-tip regression using TempEst showed a positive association between sampling time and genetic divergence in the overall *RdRp* region and *VP1* gene datasets (R^2^ = 0.8788 and 0.7979, respectively; Supplementary Figure S3A,B), indicating the presence of temporal signal. Similar positive relationships were also observed in the *VP1* gene datasets grouped as Clades 1/2 and Clades 3/4 (R^2^ = 0.4756 and 0.8159, respectively; Supplementary Figure S3C,D), although the signal was weaker in Clades 1/2 than in Clades 3/4. Overall, these results supported the suitability of the datasets for subsequent Bayesian time-scaled analysis.

### 3.3. Phylogenetic Distance Analysis

**Figure 1:**
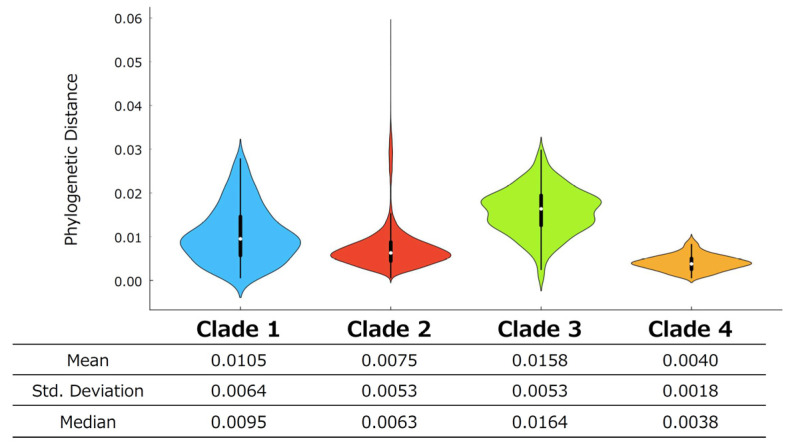
Phylogenetic distance distributions of the *VP1* gene among NoV GII.17[P17] clades illustrated using violin plots. The width of each violin represents the kernel density, indicating the distribution of phylogenetic distance values. Clade 1, Clade 2, Clade 3, and Clade 4 are shown in light blue, red, green, and gold, respectively. Black box plots indicate the interquartile range, with white dots representing median values, and whiskers showing the data range. Detailed statistical results are shown below the violin plots. Significant differences were observed among all pairwise comparisons of clades (unpaired *t*-test with Welch’s correction; *p* < 0.0001).

The genetic divergence of the *VP1* gene within each clade was evaluated based on patristic distance distributions derived from the maximum likelihood phylogenetic tree (Figure 1). The phylogenetic distance values of the *VP1* gene in NoV GII.17[P17] were 0.0105 ± 0.0064 for Clade 1, 0.0075 ± 0.0053 for Clade 2, 0.0158 ± 0.0053 for Clade 3, and 0.0040 ± 0.0018 for Clade 4 (mean ± standard deviation). Statistically significant differences were detected for all pairwise comparisons among clades using an unpaired *t*-test with Welch’s correction (*p* < 0.0001).

From a phylogenetic perspective, Clade 1 and Clade 3, which occupy relatively more ancestral positions, exhibited higher levels of genetic diversity. In contrast, Clade 2 and Clade 4, which appear to be derived from these clades, showed greater sequence similarity compared with their respective parental clades. In addition, several strains within Clade 2 displayed relatively high phylogenetic distance values. These strains were predominantly detected between 2021 and 2024 and were characterized by comparatively longer branches within Clade 2 in the phylogenetic tree.

### 3.4. Similarity Plot Analysis

**Figure 2:**
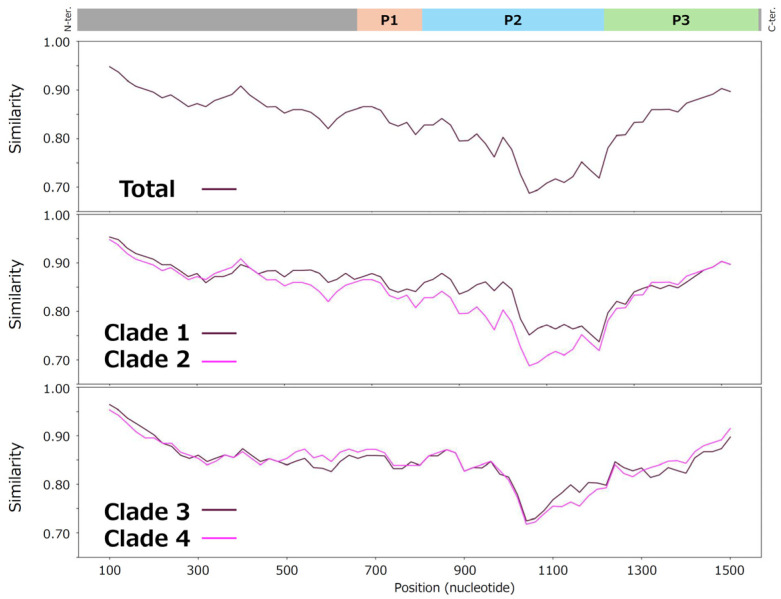
Similarity plot analysis of the *VP1* gene in NoV GII.17[P17]. Nucleotide similarity to the prototype strain (GenBank accession no. AB684681; Hu/GII/27-3/Tokyo/1976/JPN, collected in 1976) was calculated using SimPlot V1.3.

To evaluate genetic diversity within the genotype, similarity plot analysis was performed using nucleotide sequences of the *VP1* gene of NoV GII.17[P17]. A prototype strain detected in 1976 was used as the reference sequence, and analyses were conducted for all NoV GII.17[P17] strains excluding the prototype, as well as for each clade separately (Figure 2).

As a result, a reduction in sequence similarity was observed within the P domain of the *VP1* gene, particularly in the P2 domain, across all NoV GII.17[P17] strains and in each clade. In contrast, relatively high similarity was maintained in regions corresponding to the P1 and S domains. These findings indicate that high divergence sites within the *VP1* gene are predominantly localized in the P2 domain.

### 3.5. Time-Scaled Phylogenetic Analysis

**Figure 3:**
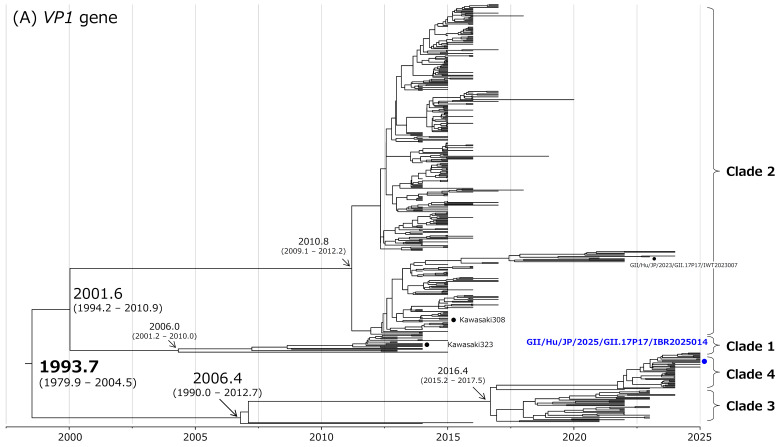

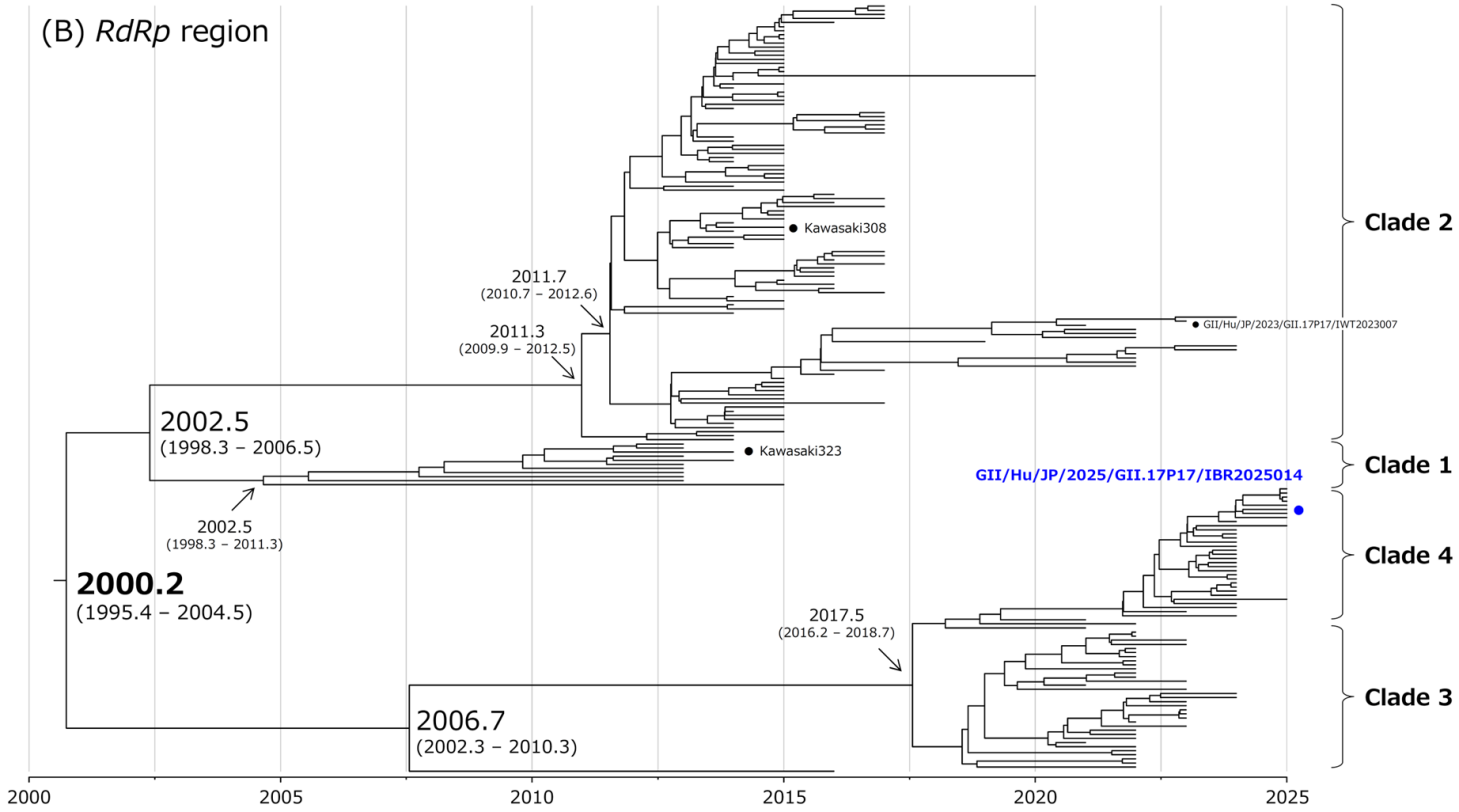
Time-scaled phylogenetic trees of the *VP1* gene (**A**) and *RdRp* region (**B**) of NoV GII.17[P17] inferred using a Bayesian MCMC framework. The horizontal axis represents time in years.

To infer the evolutionary history of NoV GII.17[P17], time-scaled phylogenetic trees were constructed based on the *VP1* gene and the *RdRp* region using a Bayesian MCMC approach implemented in BEAST2 (Figure 3A,B).

The most recent common ancestor (MRCA) of NoV GII.17[P17] was estimated to have existed around 1993.7 for the *VP1* gene (mean; 95% HPD, 1979.9–2004.5) with overlapping 95% HPD intervals and around 2000.2 for the *RdRp* region (mean; 95% HPD, 1995.4–2004.5) (Figure 3A,B). Following this ancestral divergence, the lineages split into two major branches corresponding to Clade 1 and Clade 2, and to Clade 3 and Clade 4.

Within the lineages corresponding to Clade 1 and Clade 2, additional divergence into two subclades was inferred at approximately 2001.6 for the *VP1* gene (mean; 95% HPD, 1994.2–2010.9) and 2002.5 for the *RdRp* region (mean; 95% HPD, 1998.3–2006.5). Furthermore, within Clade 2, the *VP1* gene and *RdRp* region diverged into two subclades around 2010.8 (mean; 95% HPD, 2009.1–2012.2) and 2011.7 (mean; 95% HPD, 2010.7–2012.6), respectively.

In contrast, these subclades were not clearly resolved in the maximum likelihood (ML) phylogenetic trees shown in Supplementary Figure S2. This difference likely reflects the fact that ML-based phylogenetic inference relies primarily on genetic distances among sequences without explicitly incorporating sampling dates, whereas time-scaled phylogenetic analysis explicitly incorporates sampling dates to estimate evolutionary rates and divergence times, resulting in clearer resolution of temporally structured branching patterns. As the present study focuses primarily on the recently circulating Clade 3 and Clade 4 lineages, subclades within Clade 1 and Clade 2 were not examined in further detail. Moreover, the divergence between Clade 3 and Clade 4 was estimated to have occurred around 2016.4 for the *VP1* gene (mean; 95% HPD, 2015.2–2017.5) and around 2017.5 for the *RdRp* region (mean; 95% HPD, 2016.2–2018.7), indicating that the currently circulating Clade 3 and Clade 4 strains represent a relatively recent diversification event within the Clade 3/4 lineage.

### 3.6. Phylodynamic Analysis and Evolutionary Rates Inferred Using Bayesian Skyline Plots

**Figure 4:**
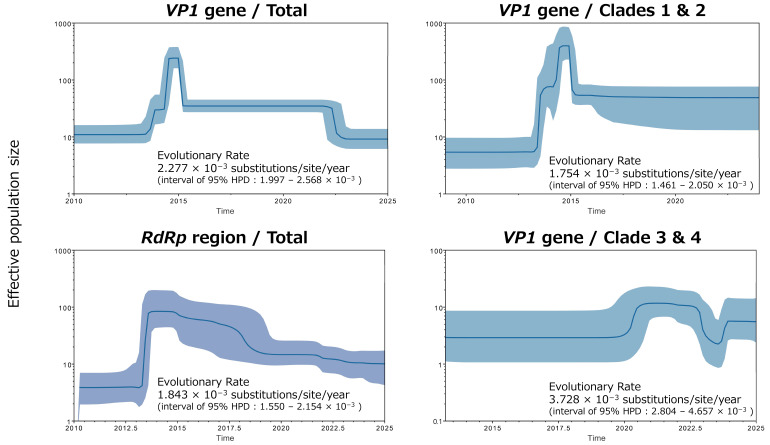
Phylodynamics and evolutionary rates of the *VP1* gene and *RdRp* region of NoV GII.17[P17] inferred using Bayesian skyline plot (BSP) analysis. The y-axis represents the effective population size on a logarithmic scale, and the x-axis indicates time in years. The blue line shows the median estimate over time, and the shaded area represents the 95% highest posterior density (HPD) interval. Estimated evolutionary rates (substitutions/site/year) with their 95% HPD intervals are shown in each plot.

Phylodynamics of NoV GII.17[P17] were inferred for the *VP1* gene and the *RdRp* region using the Bayesian skyline plot (BSP) method, and the estimated effective population sizes (EPS) are shown in Figure 4. For both the *VP1* gene and the *RdRp* region, a pronounced increase in EPS was observed after 2013. This increase coincided primarily with an expansion in the number of detected strains belonging to Clade 2. Thereafter, the EPS inferred from the *VP1* gene remained relatively stable, whereas that inferred from the *RdRp* region exhibited a gradual declining trend.

In addition, BSP analyses of the *VP1* gene were performed separately for lineages corresponding to Clade 1 and Clade 2, and to Clade 3 and Clade 4. The EPS trajectory for the Clade 1 and Clade 2 lineage was largely consistent with that observed in the analysis including all strains. In contrast, no marked fluctuations in EPS were detected for the lineage corresponding to Clade 3 and Clade 4.

Evolutionary rates of the *VP1* gene and the *RdRp* region were also estimated from the BSP analyses, and the results are summarized in Figure 4. Significant differences in evolutionary rates were detected among all comparisons (Kruskal–Wallis test, *p* < 0.0001). The mean evolutionary rate of the *VP1* gene across all strains was estimated at 2.277 × 10^−3^ substitutions/site/year, whereas that of the *RdRp* region was 1.843 × 10^−3^ substitutions/site/year. When stratified by lineage for the *VP1* gene, the evolutionary rate was estimated at 1.754 × 10^−3^ substitutions/site/year for the Clade 1 and Clade 2 lineage, compared with a higher rate of 3.728 × 10^−3^ substitutions/site/year for the Clade 3 and Clade 4 lineage. These findings suggest that, although fluctuations in EPS were relatively limited, the Clade 3 and Clade 4 lineages have accumulated genetic changes more rapidly over a shorter time period, based on the present dataset.

### 3.7. 3D Mapping of Conformational Epitopes and Positively Selected Sites in the Capsid Dimer Protein

**Figure 5:**
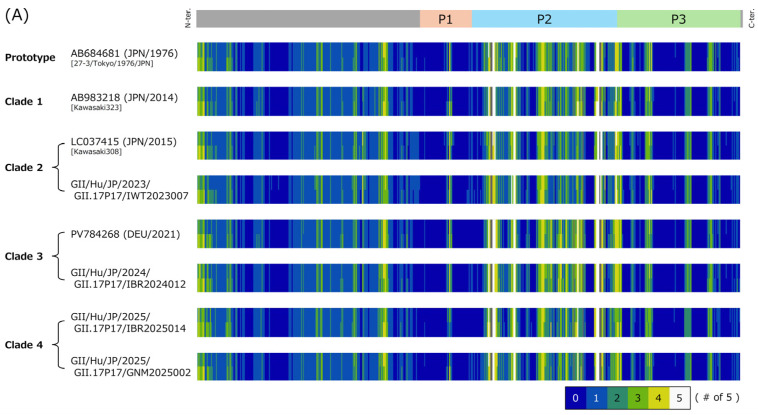

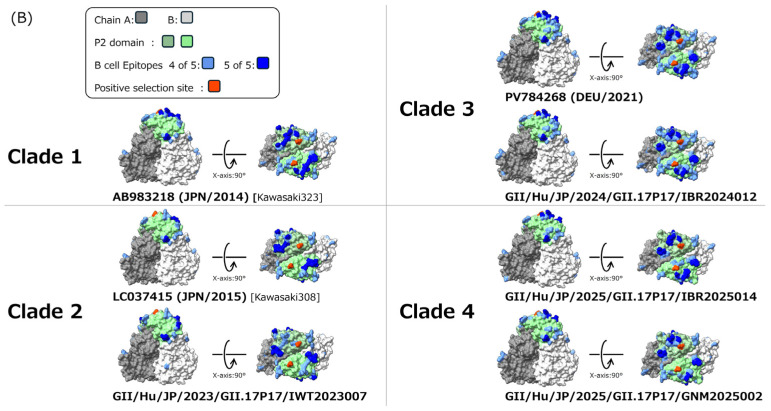
Structural mapping of predicted conformational epitopes and sites under positive selection in the capsid protein. (**A**) Heat maps showing the number of epitope prediction methods identifying each amino acid residue as a conformational B-cell epitope. Residues predicted by 0–5 of the five methods are colored dark blue, blue, dark green, green, yellow, and white, respectively. (**B**) Capsid dimer structural model. Chains A and B are shown in gray (#808080) and light gray (#D3D3D3), respectively. P2 domains on chains A and B are highlighted in sea green (#8FBC8F) and light green (#90EE90). Residues predicted as epitopes by four or five methods are shown in cornflower blue (#6495ED) and blue (#0000FF), respectively. Sites inferred to be under positive selection by all five algorithms are shown in orange-red (#FF4500).

Predicted conformational epitopes were mapped onto the 3D structural models of the capsid dimer protein for representative strains from each clade, as shown in Figure 5. To evaluate the reliability of the predicted epitope sites, five epitope prediction methods based on different theoretical frameworks were applied, and the number of methods predicting each site as an epitope was visualized as a heatmap (Figure 5A). Detailed results of the epitope prediction analyses are provided in Supplementary Table S4.

Regions predicted with high confidence as conformational epitopes were predominantly concentrated within the P2 domain of the capsid protein. Visualization of these regions on the 3D structural models of the capsid dimer revealed that the P2 domain is located at the distal tip of the capsid protrusions, where strongly predicted epitope sites were spatially clustered (Figure 5B). In addition, amino acid residues inferred to be under positive selection by all five algorithms were also located within these epitope-dense regions. In contrast, only a limited number of high-confidence epitope sites were identified outside the P2 domain. These characteristics were consistently observed across all analyzed clades, and no substantial reorganization of epitope distribution among clades was evident.

Although numerous sites inferred to be under negative selection were identified, no clade-specific or functionally distinctive distribution patterns were observed.

### 3.8. Amino Acid Substitutions in the Capsid Dimer Protein

**Figure 6:**
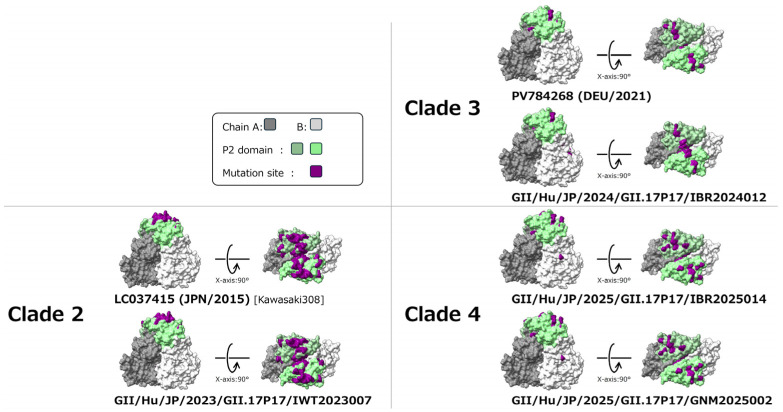
Structural mapping of amino acid substitutions in the capsid protein relative to the clade 1 representative strain Kawasaki323 (AB983218_JPN_2014). Chains A and B are colored in gray (#808080) and light gray (#D3D3D3), respectively, with P2 domains on chains A and B highlighted in sea green (#8FBC8F) and light green (#90EE90). Amino acid residues substituted relative to Kawasaki323 are shown in purple (#800080).

Figure 6 illustrates the amino acid residues that differ among clades, visualized on the 3D structural model of the capsid dimer protein, using Kawasaki323 (AB983218), a representative strain of Clade 1, as the reference sequence. Kawasaki323 (Clade 1) represents the earliest lineage among the epidemic clade group of NoV GII.17[P17] detected in Japan and constitutes a likely ancestral source from which subsequent clades emerged. This strain has been well characterized both molecularly and phylogenetically. Accordingly, Clade 1 was selected as an appropriate reference to evaluate amino acid changes accumulated in later-emerging clades. Detailed information on the amino acid substitutions analyzed in this study is provided in Supplementary Table S5.

Comparative structural mapping revealed that, in Clade 2, Clade 3, and Clade 4, amino acid residues differing from those of Clade 1 were consistently concentrated in the protruding region of the capsid, corresponding to the P2 domain. Notably, Clade 2, which is directly descended from Clade 1, exhibited a large number of amino acid substitutions within the P2 domain, suggesting a stepwise accumulation of mutations following divergence from Clade 1. In contrast, Clade 3 and Clade 4 showed fewer amino acid substitutions relative to Clade 2, and their overall amino acid differences from Clade 1 were comparatively limited. These findings suggest that, at the amino acid level, Clade 3 and Clade 4 are more closely related to Clade 1 than to Clade 2.

### 3.9. Assessment of Phylogenetic Concordance Between the RdRp Region and the VP1 Gene (Trait Analysis)

**Figure 7:**
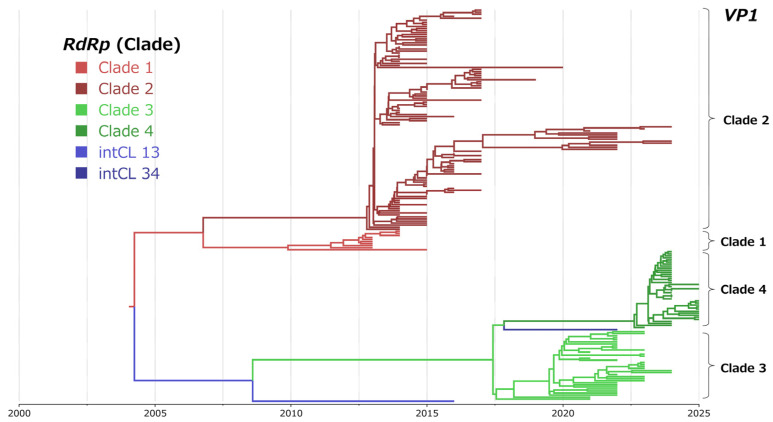
Discrete trait analysis of the *VP1* gene based on *RdRp* clade assignments. A time-scaled phylogenetic tree of the *VP1* gene is shown, with branches colored according to clade classifications inferred from the *RdRp* region. Concordant clustering of *VP1* sequences with their corresponding *RdRp* clades indicates phylogenetic consistency between the two genomic regions.

To assess the presence of potential recombination events between the *RdRp* region and the *VP1* gene, a Bayesian discrete trait analysis was performed, and the results are shown in Figure 7. In this analysis, clade assignments based on the *RdRp* region were treated as discrete traits and mapped onto the time-scaled phylogenetic tree inferred from the *VP1* gene sequences.

As a result, the phylogenetic placement of the *VP1* gene was consistently concordant with the corresponding *RdRp* clade assignments, and no phylogenetic incongruence between the two genomic regions was observed across any clade. These findings indicate that, among the NoV GII.17[P17] strains analyzed in this study, no recombination events between the *RdRp* region and the *VP1* gene that would affect the major phylogenetic structure were detected. Instead, the results support the notion that these two genomic regions have largely evolved in a coordinated manner.

### 3.10. Formal Recombination Analysis of the RdRp Region and VP1 Gene

Recombination screening using multiple methods in RDP5 did not identify any reproducible recombination signal among the analyzed GII.P17-GII.17 strains. Likewise, similarity plot and bootscan analyses using representative sequences or clade consensus sequences did not reveal any clear switching of phylogenetic affinity across the analyzed region, including the ORF1/ORF2 junction (Supplementary Figure S4). Taken together with the congruent phylogenetic topologies of the *RdRp* region and *VP1* gene, these results indicate that no detectable recombination was evident within the analyzed GII.P17-GII.17 dataset.

### 3.11. Geographic Patterns in the VP1 Haplotype Network

**Figure 8:**
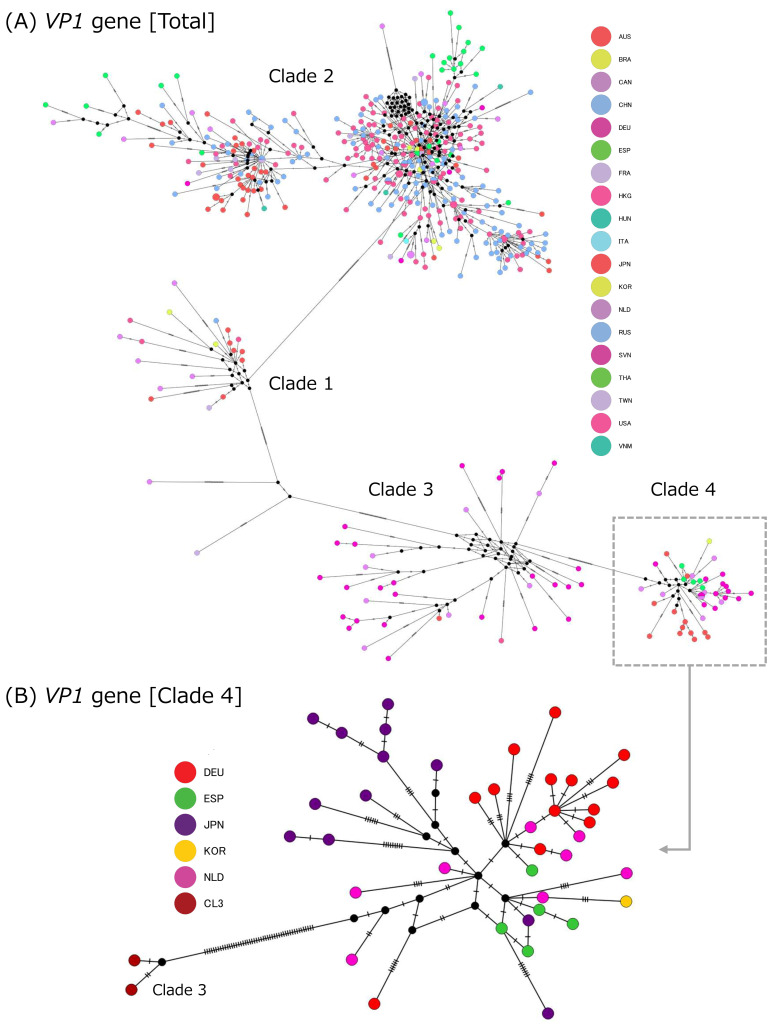
Median-joining network of VP1 haplotypes annotated by geographic origin. Median-joining network of the VP1 gene constructed using PopART. (**A**) Network including all VP1 sequences analyzed in this study. (**B**) Expanded view of the network for Clade 4. Each node represented a unique haplotype, and node size was proportional to the number of sequences sharing that haplotype. Black circles indicate median vectors, representing inferred unsampled or hypothetical intermediate haplotypes. Branches represented mutational steps between haplotypes.

Median-joining network analysis of the *VP1* gene showed that the major haplotype groups were broadly consistent with the clade structure inferred from phylogenetic analysis (Figure 8). However, in the full network, sequences from multiple countries or regions were intermingled within each major cluster, and clear geographic segregation was limited. This pattern is consistent with broad interregional distribution of the major GII.17[P17] lineages rather than confinement to a single geographic setting. At the same time, small terminal clusters enriched for a single country or region were observed in some branches, suggesting local spread after broader dissemination.

A similar pattern was observed in the Clade 4 network. Sequences from different geographic origins were distributed around shared central haplotypes, and clear country-specific subdivision was not evident. These findings indicate that the currently circulating Clade 4 is characterized by substantial geographic mixing at the *VP1* gene haplotype level.

## 4. Discussion

In this study, we comprehensively characterized the evolutionary dynamics and structural features of norovirus GII.17[P17] by integrating phylogenetic, phylodynamic, and structural analyses of the *RdRp* region and *VP1* gene. Our results consistently resolved four major clades (Clades 1–4) into two broader evolutionary lineages, Clade 1/2 and Clade 3/4, and demonstrated a clear temporal shift in predominance from the historically dominant Clade 2 to the recently circulating Clades 3 and 4. Time-scaled phylogenetic inference suggested that the Clade 3/4 lineage diverged prior to the large-scale emergence of GII.17 around 2013, indicating that its recent predominance reflects the re-emergence of a previously underdetected lineage rather than the appearance of a novel variant. The temporal signal detected by TempEst further supports the use of molecular clock-based analysis in this study, although this result should be interpreted as supportive rather than definitive evidence. Phylogenetic concordance between the *RdRp* region and *VP1* gene, supported by discrete trait analysis, further indicated coordinated evolution of these genomic regions and argued against recombination as a major driver of the observed clade structure. Comparative sequence and structural analyses showed that genetic variation was predominantly concentrated in the VP1 P2 domain, the major antigenic region of the norovirus capsid. In this context, Clade 2 underwent extensive amino acid diversification, whereas the Clade 3/4 lineage remained genetically closer to the ancestral Clade 1, suggesting distinct evolutionary strategies between the two lineages. Structural mapping revealed that predicted epitopes and positively selected sites were spatially clustered at the distal tips of the capsid protrusions, indicating strong functional constraints. Collectively, these findings support a model in which the post-pandemic predominance of GII.17[P17] was driven by lineage turnover and shifts in the immune landscape rather than by recombination or extensive antigenic remodeling. The present study was specifically designed as a genotype-focused analysis of GII.17[P17] to clarify its evolutionary dynamics. Accordingly, the circulation patterns of other norovirus genotypes in the study population were not systematically analyzed here, and such broader epidemiological comparisons were beyond the scope of the present work.

Next, the temporal trajectories of individual clades revealed marked differences in their phylogeny. Clade 2, which emerged subsequent to Clade 1, exhibited a marked expansion in effective population size and became the dominant lineage for a defined period. However, this clade subsequently declined and is no longer the main circulating lineage. In contrast, Clade 3 and Clade 4 form a distinct lineage separate from Clade 1/2, with divergence times estimated to predate the large-scale emergence of GII.17 around 2013 [44]. The limited detection of Clade 3/4 strains prior to the COVID-19 pandemic suggests that this lineage may have circulated at low prevalence, in restricted populations, or in regions with limited genomic surveillance. The recent dominance of Clade 4 therefore likely reflects not a newly emerged lineage, but rather the resurgence of a previously underdetected evolutionary branch.

The estimated evolutionary rate of the GII.17 *VP1* gene in the present study (2.277 × 10^−3^ nucleotide substitutions/site/year) was broadly consistent with a previous estimate reported for GII.17 (2.7 × 10^−3^) [16]. Compared with other major human norovirus genotypes, this rate appears to be lower than those reported for GII.2 (2.99 × 10^−3^) [11], GII.3 (4.16 × 10^−3^) [45], and especially GII.4 (7.68 × 10^−3^) [6]. The relatively high evolutionary rate of GII.4 has been linked to its rapid antigenic turnover and repeated global emergence [9,15]. At the same time, a broader comparative study across multiple human norovirus genotypes concluded that the overall rates of evolution were similar for most genotypes, ranging from 1.12 to 4.86 × 10^−3^ nucleotide substitutions/site/year, with substantial overlap in the 95% HPD intervals among many genotypes [46]. Taken together, these findings suggest that the evolutionary rate of GII.17 is not exceptional among human noroviruses overall, but may be relatively lower than that of highly dynamic genotypes such as GII.4, which could be consistent with the more constrained evolutionary pattern inferred for GII.17[P17] in the present study.

To complement the phylogenetic and phylodynamic analyses, we additionally examined the geographic distribution of *VP1* gene haplotypes using a median-joining network. This analysis showed that sequences from multiple countries or regions were widely interspersed within the major GII.17[P17] clusters, particularly within the currently predominant Clade 4. The limited geographic segregation observed in the network suggests that recent GII.17[P17] spread has involved broad interregional dissemination rather than long-term geographic isolation of distinct sublineages. The presence of small region-enriched terminal clusters may reflect localized transmission following wider spread. This analysis was intended as a descriptive and complementary assessment of geographic structure. Because median-joining networks do not provide formal estimates of ancestral location, migration direction, or introduction timing, the results should be interpreted as supplementary evidence of geographic mixing rather than definitive phylogeographic inference. Nevertheless, the network adds a useful spatial perspective to the molecular epidemiology of GII.17[P17].

Across all analyses, genetic variation within GII.17[P17] was strongly concentrated in the *VP1* gene, particularly within the P2 domain. This finding is consistent with previous studies demonstrating that the P2 domain constitutes the major antigenic region of the norovirus capsid and plays a central role in immune recognition [47,48]. Comparative analysis of amino acid substitutions revealed contrasting evolutionary strategies among clades. Relative to Clade 1, Clade 2 accumulated a large number of substitutions in the P2 domain, suggesting progressive antigenic diversification during its expansion. In contrast, Clade 3 and Clade 4 exhibited fewer substitutions and remained genetically closer to Clade 1 at the amino acid level. These observations suggest that the re-expansion of Clade 3/4 may have occurred without extensive antigenic remodeling and are compatible with the hypothesis that this lineage re-emerged while retaining ancestral antigenic features. Such a pattern may be consistent with a re-emergence driven primarily by evasion of population-level immunity shaped by prior exposure to antigenically distinct Clade 2 viruses, rather than by continuous antigenic drift, in line with the previously proposed distinction between “evolving” and “static” genotypes [49]. However, this interpretation remains hypothetical rather than definitive.

Structural mapping of predicted conformational epitopes and positively selected sites further reinforced this interpretation. High-confidence epitopes and sites under positive selection were predominantly localized to the distal tips of the capsid protrusions within the P2 domain of the capsid dimer. This region spatially overlaps with proposed receptor-binding interfaces, highlighting its functional importance in host interaction [50]. Despite clade-specific sequence differences, the overall spatial organization of epitopes was largely conserved across clades, suggesting strong structural and functional constraints. From a translational perspective, the concentration of immune-relevant and positively selected sites in a limited surface-exposed region underscores the potential of the P2 domain as a target for antiviral drug development or broadly reactive therapeutic strategies.

Moreover, our findings are broadly consistent with recent work by Tohma et al. (2025) [51], who demonstrated that norovirus capsid evolution is shaped by structural constraints and surface topology, particularly in regions involved in host interactions. The present study extends these observations by integrating long-term phylodynamic trends and clade-level evolutionary histories, providing a population-scale framework that reconciles structural conservation with lineage replacement. Together, these results support a model in which norovirus evolution proceeds through lineage turnover driven by subtle but strategically positioned mutations within antigenically and functionally critical regions.

Finally, some limitations may be acknowledged. First, the functional relevance of predicted epitopes and inferred immune escape mechanisms could not be experimentally validated, as robust neutralization assays for human norovirus remain unavailable. Consequently, the proposed role of antigenic differences in driving the re-emergence of Clade 3/4 should be interpreted as a plausible hypothesis rather than definitive proof. In addition, uneven temporal and geographic sampling may have influenced estimates of clade emergence and population dynamics. Future studies integrating expanded genomic surveillance, seroepidemiological data, and experimental analyses of receptor binding and immune recognition will be essential to further clarify the mechanisms underlying the persistence, re-emergence, and epidemiological success of GII.17[P17]. Although whole-genome sequences were available for newly generated strains, the comparative dataset retrieved from public databases contained incomplete genomes in many cases; therefore, analyses were restricted to the RdRp region and VP1 gene to maintain dataset consistency. In addition, we note that phylogeographic relationships were not formally assessed in this study. Because the available sequences were unevenly distributed across countries and sampling periods, phylogeographic reconstruction was beyond the scope of the present work. Future analyses using more geographically balanced datasets will help clarify the spatiotemporal spread of each GII.17[P17] clade.

## 5. Conclusions

In conclusion, this study clarifies the evolutionary and structural features of norovirus GII.17[P17] through integrated phylogenetic, phylodynamic, and structural analyses of the *VP1* gene and the *RdRp* region. We demonstrate that GII.17[P17] comprises four major clades forming two distinct evolutionary lineages, Clade 1/2 and Clade 3/4, and that the recent predominance of Clade 3 and Clade 4 represents a re-emergence of an evolutionary lineage phylogenetically distinct from the previously dominant Clade 2. Time-scaled phylogenetic inference suggests that this lineage diverged before the widespread emergence of GII.17 around 2013, implying prolonged low-level circulation or limited detectability before its recent expansion. Genetic diversification was largely concentrated in the VP1 P2 domain, a key antigenic region of the capsid, with Clade 3/4 remaining more closely related to ancestral strains than Clade 2. Although experimental validation of antigenicity was not feasible, these findings provide important insights into norovirus lineage turnover and highlight the value of continued genomic surveillance to better understand norovirus evolution and re-emergence dynamics.

## Figures and Tables

**Table 1:** Number of NoV GII.17[P17] strains in this study.

Gene	Total	Clade 1	Clade 2	Clade 3	Clade 4	Clade1/3 (Intermediate)	Clade3/4 (Intermediate)
*VP1*	446 (15)	19	351 (2)	34 (1)	41 (12)	2	1
*RdRp*	188 (13)	11	107 (2)	36 (1)	32 (10)	1	1 *

Numbers in parentheses indicate strains sequenced in this study. An asterisk (*) indicates that, although the strain clades within Clade 3 based on the *RdRp* region, it was classified as intermediate Clade 3/4 in this table for consistency with the *VP1*-based classification.

## Data Availability

The data presented in this study are available on request from the corresponding author.

## Supplementary Materials

The following supporting information can be downloaded at: https://www.mdpi.com/article/10.3390/microorganisms14040770/s1, Table S1: List of NoV GII.P17-GII.17 strains sequenced in this study. Table S2: List of NoV GII.P17-GII.17 strains retrieved from NCBI GenBank used in this study. Table S3: Parameters used in the Bayesian Markov chain Monte Carlo (MCMC) analyses. Table S4: Summary statistics and effective sample size (ESS) values for each parameter from the BEAST analyses. Table S5: Conformational Epitopes, Positively Selected Sites, and Amino acid substitutions in the Capsid Dimer Protein. Supplementary Figure S1: Temporal distribution of sequences analyzed in this study by year of collection. Supplementary Figure S2: Maximum likelihood (ML) phylogenetic trees of the *VP1* gene and *RdRp* region of NoV GII.17[P17]. Supplementary Figure S3: Root-to-tip regression analysis using TempEst. Supplementary Figure S4: Similarity plot and bootscan analyses of representative and intermediate GII.P17-GII.17 strains.
