# Supplementary material for: Phylogenetic Analyses of RdRp Region and VP1 Gene in Human Norovirus Genotype GII.17[P17] Variants
Source: Microorganisms. 2026 Mar 28;14(4):770. doi: 10.3390/microorganisms14040770 (PMC13119505; doi:10.3390/microorganisms14040770)
Supplement: Supplementary file 1 [file microorganisms-14-00770-s001.zip › R1_Mizukoshi_etal_Phylogenetics_GII17_SupplementaryFigures.pdf]

Supplementary data

# Phylogenetic Analyses of RdRp Region and VP1 Gene in Human Norovirus Genotype GII.17[P17] Variants

**Fuminori Mizukoshi [1,†], Yen Hai Doan [2,†], Asumi Hirata-Saito [3], Hiroyuki Tsukagoshi [4], Takumi Motoya [5], Ryusuke Kimura [4], Tomoko Takahashi [6], Yuriko Hayashi [7,8], Yuki Matsushima [9], Kei Miyakawa [10,11], Naomi Sakon [12], Kenji Sadamasu [13], Kazuhisa Yoshimura [14], Nobuhiro Saruki [15], Yoshiyuki Suzuki [16], Masashi Uema [17], Kosuke Murakami [9], Kazuhiko Katayama [18], Akihide Ryo [1], Tsutomu Kageyama [2,*] and Hirokazu Kimura [7,19,*]**

[1] Department of Bioinformatics and Integrative Omics, National Institute of Infectious Diseases, Japan Institute for Health Security, Musashimurayama-shi 208-0011, Tokyo, Japan; mizukoshi.f@jihs.go.jp (F.M.)
[2] Department of Diagnostic Testing and Technology Research, National Institute of Infectious Diseases, Japan Institute for Health Security, Musashimurayama-shi 208-0011, Tokyo, Japan; doan.y@jihs.go.jp
[3] Department of Microbiology, Tochigi Prefectural Institute of Public Health and Environmental Science, Utsunomiya-shi 329-1196, Tochigi, Japan
[4] Department of Health Science, Gunma Prefectural Institute of Public Health and Environmental Sciences, Maebashi-shi 371-0052, Gunma, Japan
[5] Department of Virology, Ibaraki Prefectural Institute of Public Health, Mito 310-0852, Ibaraki, Japan
[6] Iwate Prefectural Research Institute for Environmental Sciences and Public Health, Morioka-shi 020-0857, Iwate, Japan
[7] Department of Health Science, Graduate School of Health Sciences, Gunma Paz University, Takasaki-shi 370-0006, Gunma, Japan; hayashi@paz.ac.jp
[8] Faculty of Medical Science and Technology, Gunma Paz University, Takasaki-shi 370-0006, Gunma, Japan
[9] Department of Diagnostic Testing and Technology Research, National Institute of Infectious Diseases, Japan Institute for Health Security, Shinjuku-ku, Tokyo 162-8640, Japan
[10] Influenza Research Center, National Institute of Infectious Diseases, Japan Institute for Health Security, Musashimurayama-shi 208-0011, Tokyo, Japan
[11] Department of Microbiology, Graduate School of Medicine, Yokohama City University, Yokohama 236-0004, Kanagawa, Japan
[12] Department of Microbiology, Osaka Institute of Public Health, Osaka 537-0025, Japan
[13] Department of Microbiology, Tokyo Metropolitan Institute of Public Health, 3-24-1 Hyakunincho, Shinjuku-ku, Tokyo 169-0073, Japan
[14] Tokyo Metropolitan Institute of Public Health, 3-24-1 Hyakunincho, Shinjuku-ku, Tokyo 169-0073, Japan
[15] Gunma Prefectural Institute of Public Health and Environmental Sciences, Maebashi-shi 371-0052, Gunma, Japan
[16] Division of Biological Science, Department of Information and Basic Science, Graduate School of Science, Nagoya City University, Nagoya-shi 467-8501, Aichi, Japan; yossuzuk@nsc.nagoya-cu.ac.jp
[17] Division of Biomedical Food Research, National Institute of Health Sciences, 3-25-26, Tonomachi, Kawasaki-ku, Kawasaki 210-9501, Kanagawa, Japan
[18] Laboratory of Viral Infection Control, O¯ mura Satoshi Memorial Institute, Graduate School of Infection Control Sciences, Kitasato University, 5-9-1, Shirogane, Minato-ku, Tokyo 108-8641, Japan; katayama@lisci.kitasato-u.ac.jp
[19] Advanced Medical Science Research Center, Gunma Paz University, Takasaki-shi 370-0006, Gunma, Japan
* Correspondence: kageyama.t@jihs.go.jp (T.K.); h-kimura@paz.ac.jp (H.K.); Tel.: +81-42-561-0771 (T.K.); +81-27-386-5648 (H.K.)
† These authors contributed equally to this work.

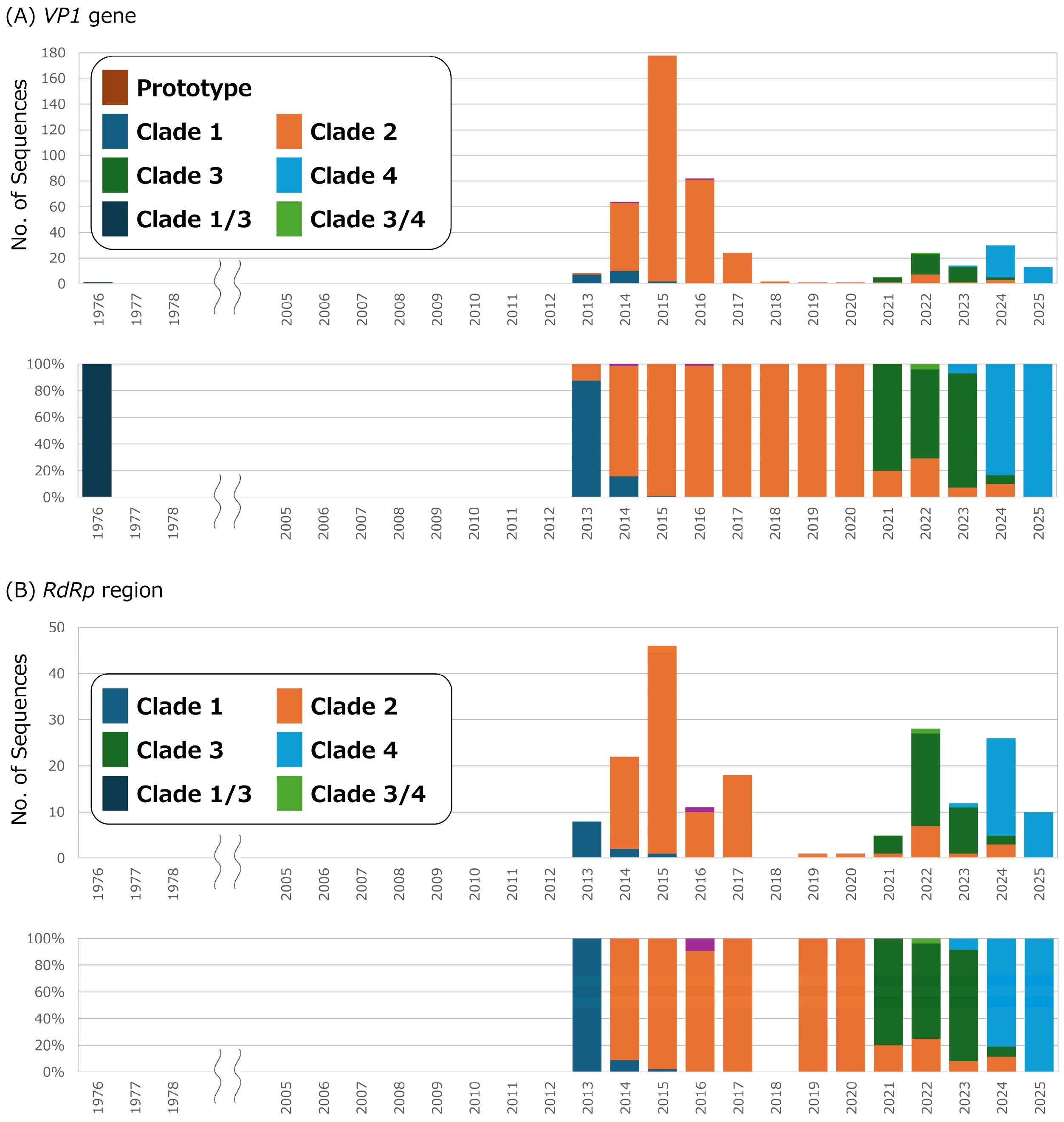


**Figure S1.** Temporal distribution of sequences analyzed in this study by year of collection. VP1 gene sequences (A) and RdRp region sequences (B) of NoV GII.17[P17] are shown.

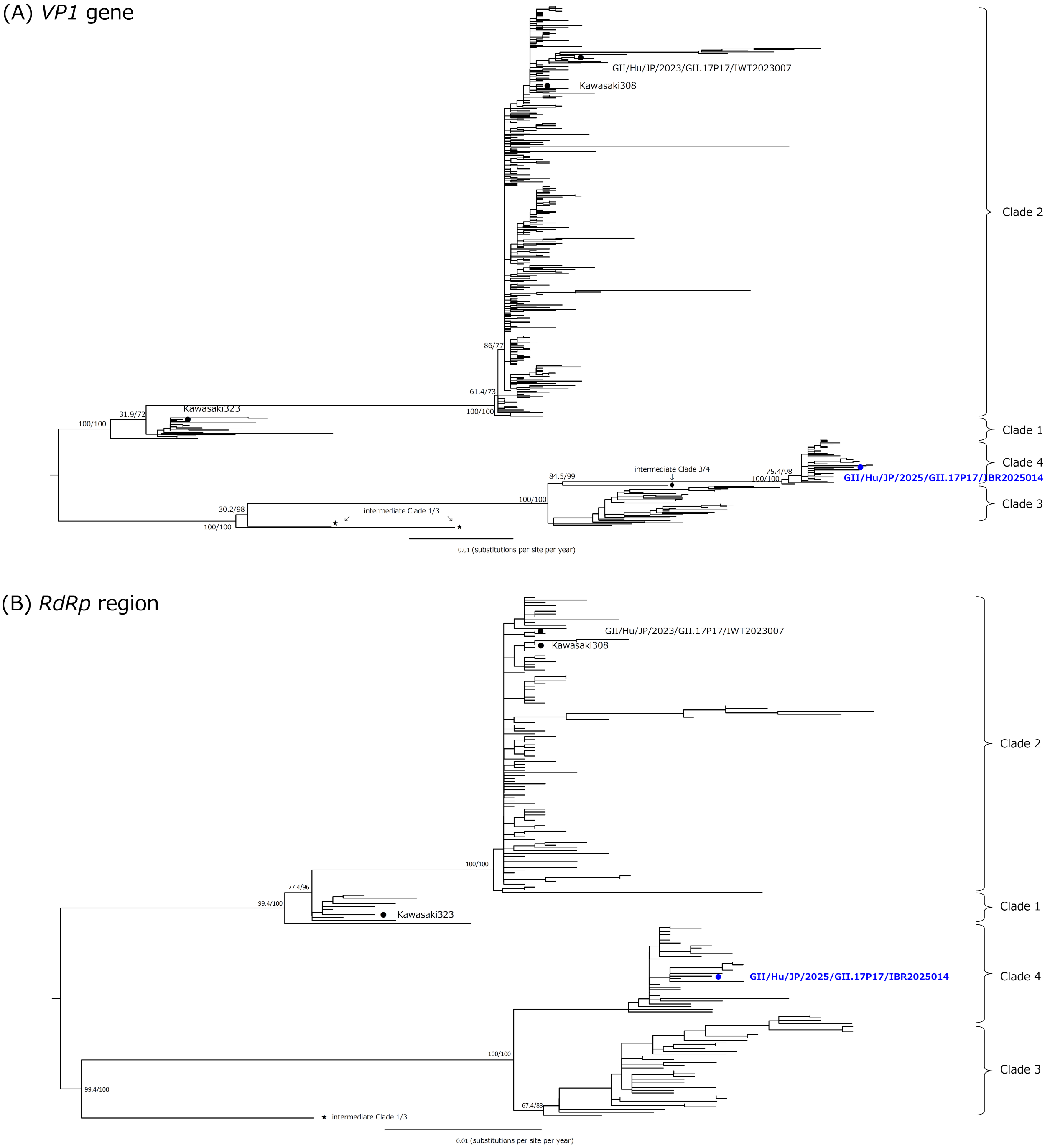


**Figure S2.** Maximum likelihood (ML) phylogenetic trees of the *VP1* gene (A) and *RdRp* region (B) of NoV GII.17[P17]. Node labels indicate SH-aLRT and ultrafast bootstrap support values. The scale bar represents nucleotide substitutions per site.

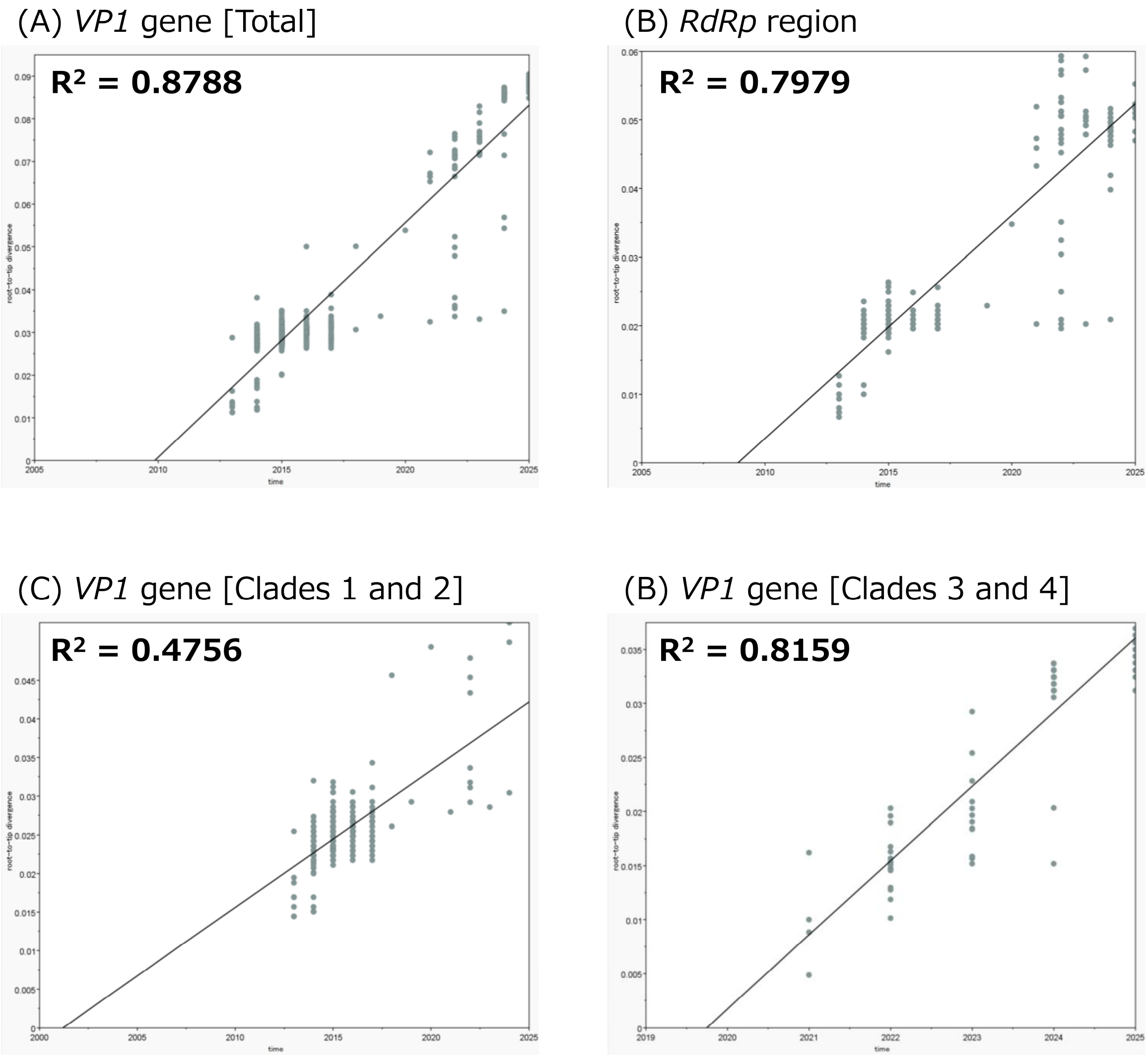


**Figure S3.** Root-to-tip regression analysis using TempEst. Temporal signal was assessed by root-to-tip regression using TempEst. The relationship between sampling time and root-to-tip genetic divergence is shown for the overall VP1 gene dataset (A), the overall RdRp regein dataset (B), the VP1 gene dataset grouped as Clades 1 and 2 (C), and the VP1 gene dataset grouped as Clades 3 and 4 (D). Each point represents one sequence, and the line indicates the fitted regression. The corresponding $R^2$ values are shown in each panel.

## (A) Similarity plot

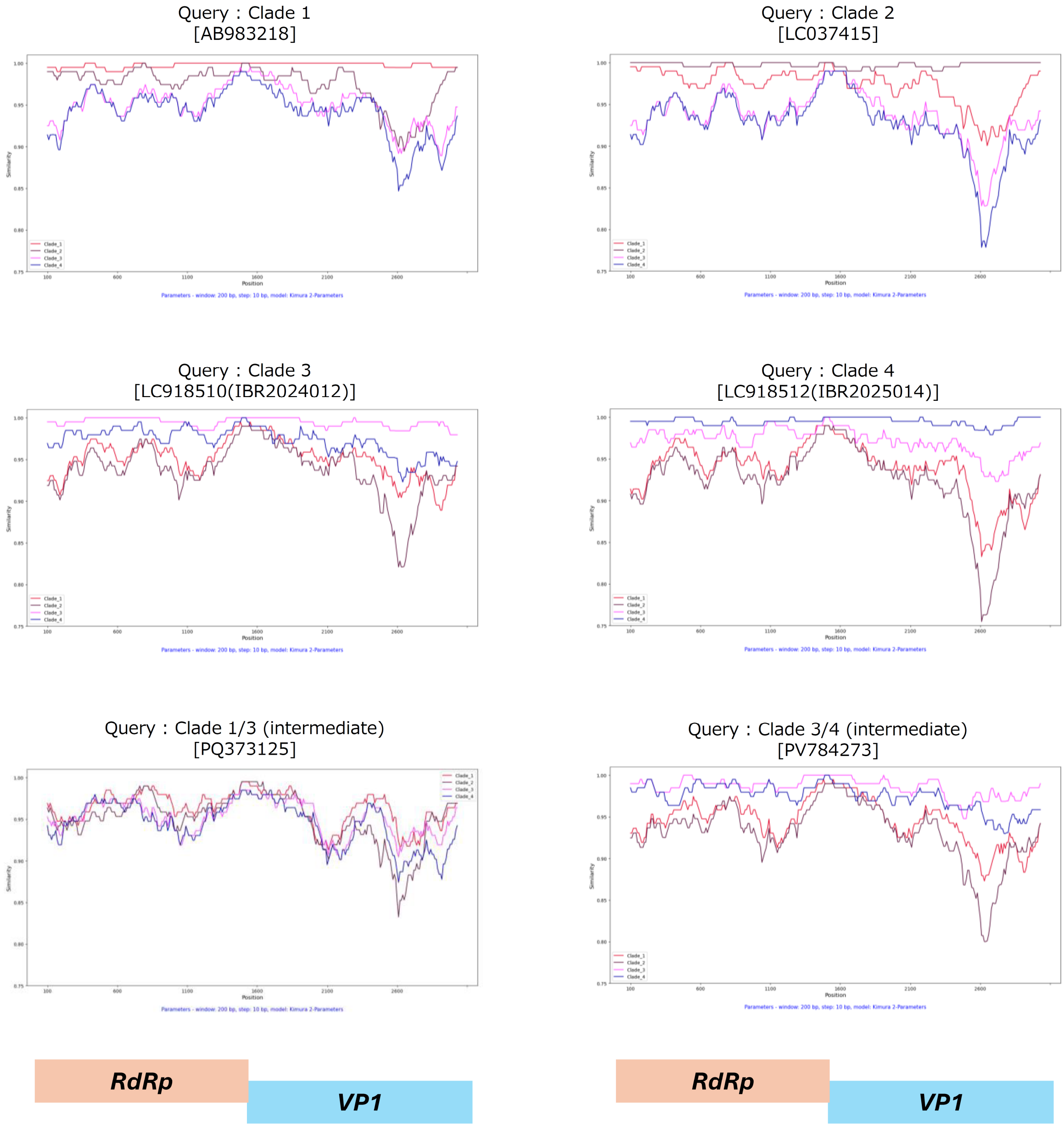

## (B) Bootscan analyses

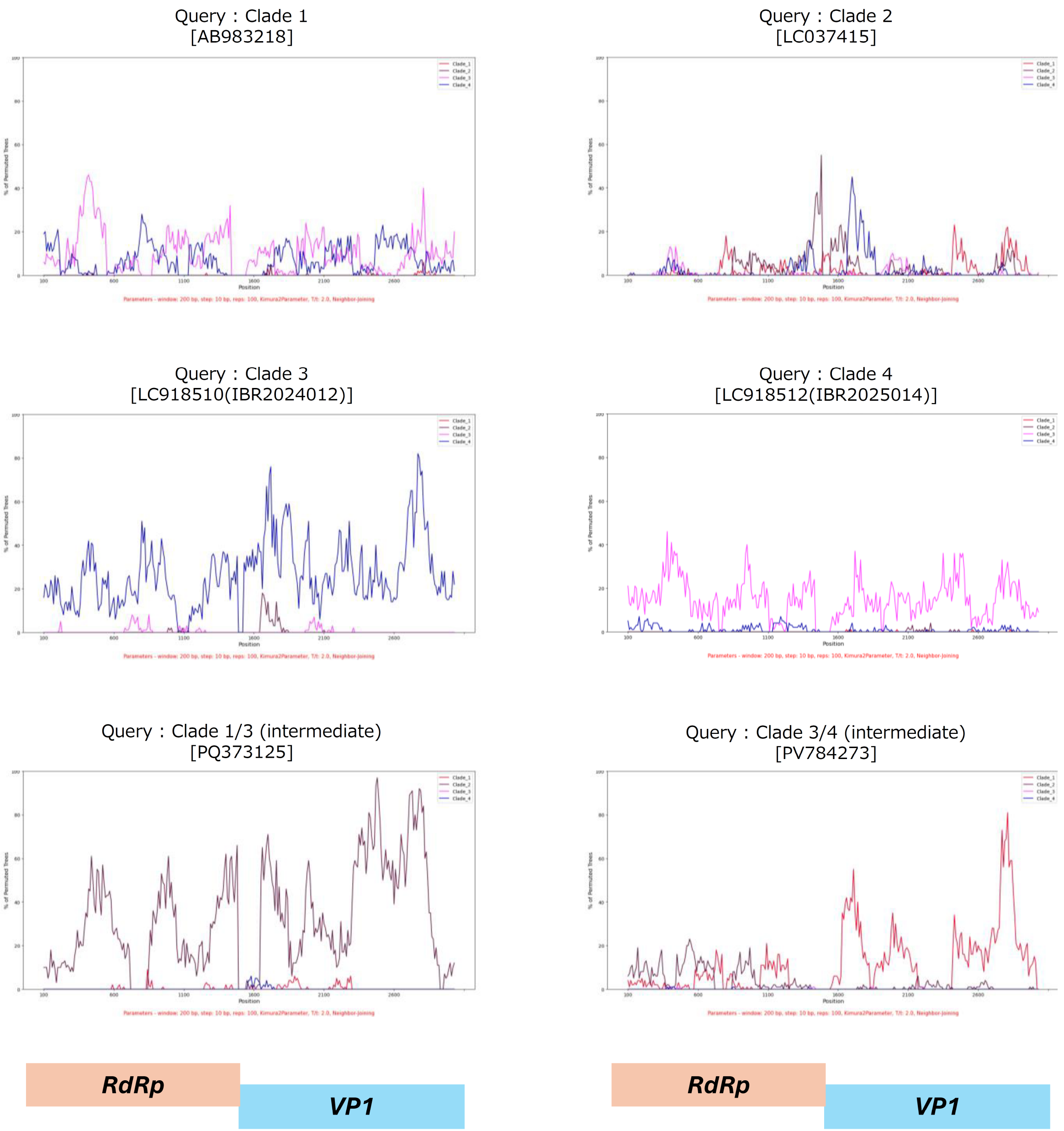


**Figure S4.** Similarity plot and bootscan analyses of representative and intermediate GII.P17-GII.17 strains. Similarity plot (A) and bootscan analyses (B) were performed for six query strains, including four representative strains from Clades 1–4 (CL1_AB983218_JPN_2014, CL2_LC037415_JPN_2015, CL3_IBR2024002_JPN_2024, and CL4_IBR2025002_JPN_2025) and two intermediate strains (intCL13_PQ373125_NLD_2016 and intCL34_PV784273_DEU_2022). Clade consensus sequences of Clades 1–4 were used as references, and the query sequence itself was excluded from the consensus of the corresponding clade. (A) Similarity plot analysis was conducted using a 200-bp sliding window and 10-bp step size under the Kimura 2-parameter model. (B) Bootscan analysis was performed with a 200-bp window, 10-bp step size, neighbor-joining trees, 100 bootstrap replicates, and a transition/transversion ratio of 2.0. Across all representative and intermediate strains, no clear and sustained crossover pattern was observed, and no reproducible shift in clade affinity was detected across the ORF1/ORF2 junction.
